# Current state of the imaging physics workforce and financial model

**DOI:** 10.1002/acm2.13489

**Published:** 2021-12-10

**Authors:** David W. Jordan, Wayne D. Newhauser, Michael D. Mills

**Affiliations:** ^1^ Department of Radiation Safety University Hospitals Cleveland Medical Center Cleveland Ohio USA; ^2^ Department of Radiology Case Western Reserve University Cleveland Ohio USA; ^3^ Department of Physics and Astronomy Louisiana State University Baton Rouge Louisiana USA; ^4^ Department of Radiation Oncology University of Louisville Louisville Kentucky USA

Nearly every citizen depends, directly or indirectly, on the services of medical physicists to ensure high‐quality and safe medical care. Medical physicists have a large societal impact, like those of engineers and teachers. In contrast to these professions, the number of medical physicists is tiny (about 9000 in the USA) and our knowledge of the profession's characteristics is incomplete.^1^ Multiple publications have examined supply and demand, workforce characteristics, and staffing levels for qualified medical physicists (QMPs) (defined as per https://www.aapm.org/org/policies/details.asp?id=449) specializing in radiation therapy (e.g., see Mills et al. https://doi.org/10.1120/jacmp.v11i2.3005; https://www.astro.org/Patient‐Care‐and‐Research/Patient‐Safety/Safety‐is‐no‐Accident; and Swanson https://doi.org/10.18297/etd/3224). At present, these details are lacking for diagnostic imaging physics and nuclear medicine physics. This is probably because about 75% of medical physicists work in therapy. As we study the imaging physics workforce, it is worthwhile to step back and consider how the current workforce serves the public interest and how the profession should monitor and manage it in the future. In this column, we appeal to our colleagues to contemplate how the future workforce may evolve from its present state.

Consider differences in financial models for radiation oncology physics and diagnostic imaging physics in the USA. Many of the clinical services provided by therapy physicists are associated with current procedural terminology codes and are eligible for reimbursement (e.g., from health insurance entities). In this direct reimbursement model, therapy physics is a revenue center and staffing models are directly linked to patient volume and revenue. In contrast, diagnostic physics services are typically treated as an overhead model. That is to say, diagnostic physics services are not eligible for direct reimbursement and are only indirectly linked directly to patient volume and revenue. With the overhead model, staffing models are largely driven by compliance with minimum requirements set by regulations and clinical accreditation programs.

Thus, differences in financial models can influence physicist staffing. The financial models may or may not be sufficient to provide optimal care to every patient. Research is needed to quantify the incremental improvements in patient care that can result from additional imaging physics support once the minimum requirements are met. The literature on diagnostic errors does not directly link diagnostic medical physics effort to outcomes, but it does show that applications with exacting image quality requirements, such as mammography and other cancer detection exams, are responsible for a large proportion of errors leading to suboptimal patient outcomes. Given the frequency of errors in diagnostic radiology procedures (14–15 diagnostic error malpractice claims per 1000 person‐years) and the potential severity of associated outcomes (42% of claims involving high‐severity injury and patient death), it is reasonable to posit that medical physicists will play essential roles in improving patient outcomes in at least some cases.^2^, ^3^, ^4^, ^5^

The American Association of Physicists in Medicine's (AAPM's) most recent published assessments of the diagnostic physics workforce are outdated (see discussion in AAPM Report 301: https://doi.org/10.37206/163). Ongoing efforts, including a survey completed in 2020 and the development of models and future projections, will provide a current snapshot of the workforce and marketplace for clinical imaging physics services. Some preliminary data are available that will be fully detailed in forthcoming AAPM publications.

We can estimate the current size of the US diagnostic physics workforce from data sources like the AAPM member database and directories of QMPs. While it is impossible to reliably predict the future workforce size, it is possible to estimate the future supply of QMPs, which is governed by the integral capacity of medical physics graduate programs and imaging physics residency programs. One of the most pressing questions for our profession is whether these programs are “right‐sized” to meet the demand for imaging physicists now and in the future.

Demand is harder to predict. The most recent projection of the future demand and required residency capacity in imaging was an AAPM‐sponsored study in 2009.^6^ The projection of required imaging physics residency capacity by 2020 was about 30 graduates per year, and there are now 35 Commission on Accreditation of Medical Physics Education Programs‐accredited imaging programs. However, forthcoming AAPM publications will report that over the same period, the total workforce size grew much faster than projected. Our profession has at its disposal historical trends, surveys, and demographics of the current workforce, and these can inform projections of attrition. Despite the known difficulties, our profession can and should project demand more frequently and with more attention to each of the major subspecialties, including diagnostic imaging physics.

The task of projecting the size of the diagnostic physics workforce would be a rather neat problem if all inflows and outflows of practicing diagnostic physicists could be identified and measured. For example, inflows include both certified and non‐Board‐certified medical physicists (NCMPs). Accreditation bodies (such as the American College of Radiology and Joint Commission) and some state regulatory agencies grant independent practice authorization to NCMPs who meet education and experience requirements. Since these individuals qualify via a variety of pathways, there is an unknowable number of inflows that are impossible to monitor. It makes sense to assume that where allowed, any shortage of board‐certified physicists would be made up, at least in part, by NCMPs in response to the excess demand.

In addition, medical physicist assistants (MPAs) in imaging physics have existed, informally, for a long time. Medical physics practice guidelines have clarified the medical physics scope of practice (Clements et al. https://doi.org/10.1002/acm2.12469) and the role of the MPA (Seibert et al. https://doi.org/10.1002/acm2.12774). With appropriate supervision by QMPs, MPAs can be effective to extend the workforce. To our knowledge, MPAs are not yet included in tallies of the imaging physics workforce. The formal definition of the MPA role is recent and, in the future, it will be important to take into account MPAs’ impact on the capacity of the imaging physics workforce.

There is anecdotal evidence of a current shortage of certified diagnostic imaging physicists. For example, numerous NCMPs currently practice on a permanent basis, outnumbering those who are temporarily an NCMP between completion of training and Board certification. Employers report that imaging physicist job postings (whether they require board certification or not) take a long time to fill, elicit smaller than desired pools of applicants and that candidates typically receive multiple competitive offers. Published survey results from 2004^7^ and unpublished AAPM survey data from 2012 and 2020 consistently show about 30% of respondents who primarily specialize in radiation oncology are providing diagnostic imaging support part‐time. It is possible that a shortage of diagnostic imaging physicists is a consequence of hyper‐specialization of professional certification requirements (see Mills, https://doi.org/10.1002/acm2.12781), which is a type of measurement bias in workforce assessments. Taken together, these and other available data suggest that a shortage of imaging physicists may exist today.

If a true shortage exists, the profession is obligated to understand why and to take corrective measures. To this end, it must be ascertained if the shortage is real (i.e., patients’ interests are not being fully met) or an artifact (e.g., caused by measurement bias). When a shortage in a profession occurs, it is incumbent upon the profession to determine its own causative role, if any. Specifically, professions enjoy title protection, limited monopoly, and power to regulate who may enter the profession. These privileges, if exercised imperfectly, can cause shortages. Hence, we must determine if the apparent shortage is real, and if so, what role we played in its creation.

There is always some degree of turnover in existing positions as individuals seek new opportunities for a variety of personal and professional reasons. A review of current or recent job postings will show a large number of diagnostic physics job postings, but it is difficult to determine which posts are net‐new positions and which result from a sort of “musical chairs” among current physicists. Even if all job postings clearly indicated whether they were for new or replacement positions, job vacancies are a lagging indicator for demand.

First‐order demand growth results from increases in factors such as the number of machines and facilities supported, exams performed, staff trained, and compliance with regulations, accreditations, registrations, and licenses maintained. At a minimum, these increases require more Level‐1 services, in the context of the levels of service model described in AAPM Report 301. We should expect such demand changes to be roughly linear, with new growth occurring predictably with new facilities and equipment. A simple demand model based on population would likely encompass first‐order demand growth on a regional or national basis.

Second‐order demand growth is more difficult to predict. Second‐order demand change results when there is a change in the number of imaging physicists needed per basis unit of service, that is, per exam, machine, or facility. This can occur when a facility with established diagnostic medical physics support seeks additional services, such as the creation and implementation of new programs or services, voluntary auditing, optimization, and accreditation programs, and so forth. Second‐order demand growth also encompasses the implementation of new mandatory requirements that were previously voluntary. Aging of the population and changing disease prevalence can increase the imaging utilization per patient in the population, in turn altering the per‐patient demand for physics support. Research is an essential activity. Research and development may impact first and/or second‐order demand; for example, it may be optional for patient care but required for an institution to be a National Cancer Institute‐designated Cancer Center.

Second‐order growth is likely to result when there is increased adoption of Level‐2 and Level‐3 services. Voluntary demand for additional medical physics support (and financial support to pay for it) would likely increase when it can be shown to produce tangible benefits to the organization, such as improved patient outcomes or access, reduced costs, increased revenue, or elevated patient or physician satisfaction. AAPM's workforce assessment efforts are studying the current level of such voluntary demand, but anecdotally, it appears quite low.

Throughout this discussion, we implicitly include nuclear medicine in diagnostic or imaging physics. As the smallest medical physics subspecialty, nuclear medicine physics comprises just a few hundred QMPs in the USA (see Harkness et al. https://doi.org/10.1120/jacmp.v16i5.5661). There is a great deal of overlap with other specialties. Many diagnostic physicists support nuclear imaging, and the emerging practice of radiopharmaceutical therapy includes many therapy physicists, who provide essential support where no nuclear medicine specialist is available. The nuclear medicine physicist's expertise in the safe handling and use of radioactive material qualifies them as a radiation safety officer, but this does not drive demand for nuclear medicine physicists since most medical RSOs come from other backgrounds in medical or health physics. The recent rapid growth of imaging and therapeutic radiopharmaceutical applications may create a rapid increase in demand for nuclear medicine physicists, and it is unclear whether the current education and training pipeline would be able to fulfill such demand with QMPs.

The current US workforce appears to have a shortage of QMPs specializing in imaging physics. The shortage appears to be ameliorated to some extent by NCMPs, MPAs, and medical physicists from other subspecialties. We must prepare for the possibility that growth and aging of the population, or increased utilization of imaging in medicine, will exacerbate shortages of imaging physicists. This is especially urgent for QMPs because of the long time required for their education, training, and professional board certification. More broadly, we need to fully utilize limited supplies of QMPs, MPAs, NCMPs, engineers, and other related professionals (e.g., radiologists) to ensure that the needs of physicians and patients for safe and high‐quality imaging are met. Most importantly, our profession must better understand current and future needs for our services and, correspondingly, ensure that adequate numbers of imaging physics workers enter our profession.

## AUTHOR CONTRIBUTIONS

All authors contributed equally to the drafting and revision of the manuscript.

## CONFLICT OF INTEREST

All authors have no conflicts of interest to disclose.
